# A Manual of Dissection and Practical Anatomy, Founded on Gray and Gerrish

**Published:** 1903-06

**Authors:** 


A Manual of Dissection and Practical Anatomy, Founded on Gray and Gerrish.—By William Eckley, M. D., Professor of Anatomy in the Medical and Dental Departments of the University of Illinois, the Chicago School of Anatomy and Physiology, the Chicago Clinical School, and West Side Training School for Nurses; and Corinne B. Eckley, Demonstrator of Anatomy in the Medical and Dental Departments of the University of Illinois, and Chicago School of Anatomy and Physiology, and Member of the Association of American Anatomists. Illustrated with 220 engravings, 116 of which are colored. Lea Bros. & Co., Philadelphia and New York.

This volume deserves commendation for the manner in which an immense amount of matter has been acceptably condensed into surprising small space. The printing is rather fine, but this detracts nothing from the value of the work, since the type and paper are excellent. The book amply fulfills its two-fold purpose, namely, to provide the student with a detailed guide for dissection, and to answer the requirements of the physician and surgeon wishing to review in a limited time the anatomy of any given region.

For a busy surgeon who wishes to review quickly the dissection of any part, previous to an operation, this book is especially serviceable, and, in most cases, is all that will be needed. The illustrations are especially good. T. P. L.

